# New Mitogenomes of the Green Lacewing Tribe Ankylopterygini (Neuroptera: Chrysopidae: Chrysopinae) and Phylogenetic Implications of Chrysopidae

**DOI:** 10.3390/insects14110878

**Published:** 2023-11-14

**Authors:** Shuo Tian, Yunlan Jiang, Yan Lai, Shutong Wang, Xingyue Liu, Yuyu Wang

**Affiliations:** 1 College of Plant Protection, Hebei Agricultural University, Baoding 071001, China; shuotian139@126.com (S.T.); jiangyunlan@hebau.edu.cn (Y.J.); bdstwang@163.com (S.W.); 2 Department of Entomology, China Agricultural University, Beijing 100193, China; lyzb152@126.com

**Keywords:** Chrysopidae, mitochondrial genome, phylogeny, divergence time

## Abstract

**Simple Summary:**

Chrysopidae are the second largest group of Neuroptera. Their larvae can prey on various agricultural and forestry pests. In this study, we sequenced two mitochondrial genomes (mitogenomes) of *Ankylopteryx* in Ankylopterygini for the first time. Comparative analyses of the mitogenomes of Chrysopidae were conducted. The arrangements of the mitogenomes were consistent with the other Chrysopidae species. The phylogeny of Chrysopidae was reconstructed and the divergence time within Chrysopidae was estimated. Chrysopinae were recovered as the sister group to Apochrysinae + Nothochrysinae. Within the subfamily of Chrysopinae, Nothancylini were recovered as the sister group to (Leucochrysini + Belonopterygini) + (Ankylopterygini + Chrysopini). The three extant subfamilies of Chrysopidae diverged from each other during the Early Cretaceous. Nothancylini diverged from other Chrysopinae in the Early Cretaceous. Leucochrysini diverged from Belonopterygini in the Late Cretaceous. Ankylopterygini diverged from Chrysopini in the Middle Cretaceous.

**Abstract:**

Chrysopidae (green lacewings) are a cosmopolitan and species-rich family of Neuroptera, with remarkable significance of biological control against various agricultural and forestry pests. However, the phylogenetic position of Chrysopidae in Neuroptera and the internal relationships within the family remain equivocal among previous studies based on different types of data and sampling. Here we sequenced the mitochondrial genomes (mitogenomes) of two species of the genus *Ankylopteryx* in the chrysopine tribe Ankylopterygini for the first time. The characteristics of these mitogenomes were analyzed in comparison with other green lacewing mitogenomes published to date. In the phylogeny herein reconstructed based on mitogenomes, Chrysopinae were recovered as the sister group to Apochrysinae + Nothochrysinae. Within the subfamily of Chrysopinae, Nothancylini were recovered as the sister group to (Leucochrysini + Belonopterygini) + (Ankylopterygini + Chrysopini). The divergence time estimation suggested an Early Cretaceous initial divergence within the extant Chrysopidae. Within Chrysopinae, the four tribes except Nothancylini diverged around mid-Cretaceous.

## 1. Introduction

Chrysopidae are the second largest group of Neuroptera, with over 1400 species from 82 genera, widely distributed around the world [1,2]. The green lacewing larvae prey on various soft-body terrestrial arthropods (e.g., aphids, mealy bugs, thrips and others), playing an important role in the biological control of agricultural and forestry pests [3,4].

Chrysopidae are composed of three extant subfamilies (Apochrysinae, Nothochrysinae and Chrysopinae) and an extinct subfamily (Limaiinae) [5]. However, the phylogenies at subfamilial and tribal levels within Chrysopidae were not exactly clear. Nothochrysinae were recovered as the sister group to Apochrysinae + Chrysopinae based on morphological characters [6]. Apochrysinae were recovered as the sister group to Nothochrysinae + Chrysopinae based on morphological characters and some molecular markers (*cox1*, *16s* rDNA, *cad* and mitogenomes) [7,8,9]. However, Chrysopinae were recovered as the sister group to Apochrysinae + Nothochrysinae based on nuclear genes and mitogenomes [10,11].

Among the five tribes, i.e., Nothancylini, Leucochrysini, Belonopterygini, Ankylopterygini and Chrysopini of Chrysopinae, Nothancylini were recovered as the sister group to (Belonopterygini + Leucochrysini) + (Chrysopini + Ankylopterygini) based on morphological data and mitogenomes [2,12,13]. Chrysopini were recovered as the sister group to Belonopterygini + (Leucochrysini + Ankylopterygini) based on some molecular data (*cad*, *cox1* and *16s* rDNA) [8,11].

In recent years, many new data and methods have been used to resolve the phylogenetic relationships within Chrysopidae. In a recent study based on molecular data (*16s* rDNA, *cox1*, CPSase, *cad*, *wg*, *pepck*, *atpase*, *18s* and mitogenomes), the Bayesian inference (BI) suggested Apochrysinae was the sister group to Nothochrysinae + Chrysopinae based on the molecular supermatrix, while the tree of maximum likelihood (ML) supported Chrysopinae was the sister group to Nothochrysinae + Apochrysinae [14]. Nothochrysinae were recovered as paraphyletic based on the anchored hybrid enrichment (AHE) data with a large sample [15]. Leucochrysini were recovered as sister group to Belonopterygini, but Chrysopini were recovered as paraphyletic in these two studies [14,15]. Then, Chrysopinae were recovered as the sister group to Apochrysinae + Nothochrysinae based on the low-coverage whole genome of five species representing all three subfamilies, and Chrysopini were recovered as the sister group to Belonopterygini [16]. However, Apochrysinae were recovered as the sister group to Nothochrysinae + Chrysopinae based on the morphological characters of genitalia and wing-venation as well as some molecular data (*16s* rDNA, *cox1*, CPSase, *cad*, *wg*, *pepck*, *atpase*, *18s* and mitogenomes) [17]. In addition, Nothancylini were recovered as the sister group to the remaining Chrysopinae (Belonopterygini + Leucochrysini) + (Ankylopterygini + Chrysopini) [17]. Herein, the higher phylogeny within Chrysopidae is still controversial.

There are eleven mitogenomes of Chrysopidae published in GenBank (http://www.ncbi.nlm.nih.gov (accessed on 11 June 2023)), but there is only an incomplete representative mitogenome of Ankylopterygini. In this study, we newly sequenced the mitogenomes of two species of the genus *Ankylopteryx* (Schneider, 1815) in Ankylopterygini. These two mitogenomes were annotated and uploaded to GenBank with accession numbers OQ269716 and OM510943. Comparative analyses of the mitogenomes of Chrysopidae were conducted here, such as codon usages, the rates of nucleotide substitution and secondary structure predictions of RNAs. The phylogeny of Chrysopidae was reconstructed based on mitogenomes with addition of the newly sequenced *Ankylopteryx* data, and the divergence time among major lineages within Chrysopidae was estimated.

## 2. Materials and Methods

### 2.1. Sampling and Genomic DNA Extraction

The specimen of *Ankylopteryx octopunctata* (Fabricius) was collected by Hongyu Li on 30 July 2018, in Jiangxin, Medog, Xizang, China and that of *A. gracilis* (Nakahara) was collected by Qicheng Yang on 10 June 2016, in Baisha River, Hekou, Yunnan, China. Both the specimens were preserved in 95% alcohol at −20 °C before the DNA extraction. Genomic DNA was extracted using the DNeasy Blood and Tissue kit (QIAGEN, Hilden, Germany) from the thoracic muscle tissue. The DNA concentration was measured using a Nucleic acid protein analyzer (Thermo Scientific, Waltham, MA, USA).

### 2.2. Genome Sequencing and Analysis

Whole genomes were sequenced on the Illumina HiSeq 2500 Platform (San Diego, CA, USA) by Majorbio (Shanghai, China). Raw reads were checked by FastQC 0.11.9 [18] and low-quality reads were filtered using Trimmomatic 0.32 [19]. The mitogenomes were assembled by IDBA-UD 1.1.3 [20]. Annotations were conducted by MitoZ 2.4 [21] with the “invertebrate mt code 5” as genetic code and “Arthropoda” as clade. Then, the sequence was checked by manual proofreading according to relative species. The circular maps of mitogenomes were drawn by OGDRAW [22], and the base composition and codon usage were analyzed using MEGA 7.0 [23]. The calculation formulas of base composition asymmetry were AT-skew = (A − T)/(A + T) and GC-skew = (G − C)/(G + C) [24]. The relative synonymous codon usage (RSCU) of protein-coding genes (PCGs) was calculated using MEGA 7.0 [25]. Sequences of thirteen PCGs of all Chrysopidae (including Genbank and the two newly sequenced mitogenomes) were individually aligned via the L-INS-i algorithm using MAFFT 7.313 [26] with the “invertebrate mitochondrial” as code table, the “codon” as alignment mode and the “auto” as strategy. Then, nucleotide diversity (Pi) and non-synonymous/synonymous substitution ratios (Ka/Ks) were calculated by DnaSP 6.12.0391 [27] with the “first site” as protein coding regions and the “mtDNA *Drosophila*” as genetic code. The secondary structures of tRNAs were predicted by the MITOS Web Server (http://mitos.bioinf.uni-leipzig.de/index.py, accessed on 25 May 2023) and checked through manual proofreading [28], and the secondary structures of *rrnS* and *rrnL* were predicted using RNA Structure (http://rna.urmc.rochester.edu/RNAstructureWeb/ (accessed on 27 May 2023)) [29].

### 2.3. Phylogenetic Analysis

**Figure 1:**
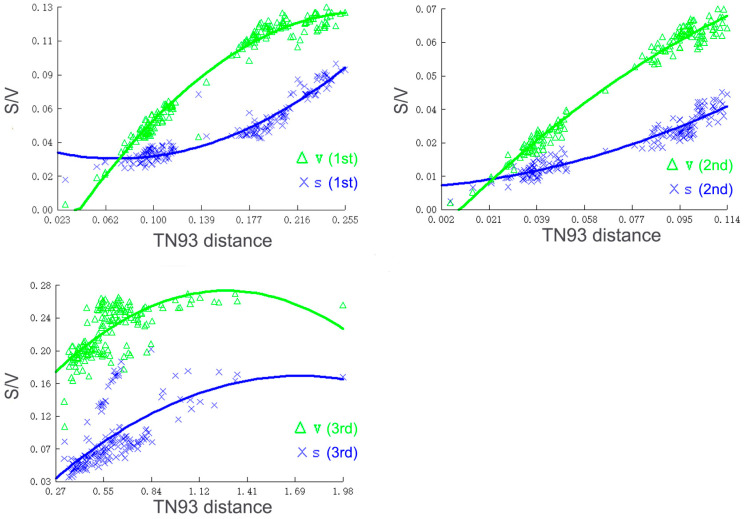
Saturation curves of different codon position transitions (S) and transversions (V) per TN93 distance.

There were thirteen species of Chrysopidae used as ingroups and two species of *Hemerobiidae*, two species of *Mantispidae* as well as two species of Myrmeleontidae were used as outgroups to reconstruct the phylogeny in this study (Table S1 [12,13,30,31,32,33,34,35,36]). Sequences of PCGs and rRNAs were aligned using MAFFT 7.313 [26]. Each rRNA gene alignment was conducted using the G-INS-i algorithm by MAFFT 7.313 [26] with the “invertebrate mitochondrial” as code table, “normal” as alignment mode and “auto” as strategy. About 0.6% of the unaligned sites were excluded using Gblocks [37] setting “with half” as allowed gap positions and default for other parameters. The dataset PCG123RNA contains 13,128 sites including all codon positions of 13 PCGs and the two rRNAs. The dataset PCGAA contains 3696 sites including all amino acids of 13 PCGs. The saturation of different codon positions was assessed using DAMBE [38,39]. Due to the heterogeneity of mitogenome composition and the third site saturation (Figure 1), the phylogenetic relationships within Chrysopidae could not be solved using single homogeneous model [33]. Heterogenous model (i.e., CAT-GTR) had been proven efficient to better solve the phylogenetic problems based on mitogenomes in many studies [40,41,42]. The phylogenetic topologies under heterogeneous models were reconstructed with BI and ML methods. The BI trees were inferred based on datasets PCG123RNA and PCGAA under the heterogeneous model CAT-GTR using PhyloBayes 3.3 [43]. Two strands ran at the same time for separate analysis until the maxdiff was less than 0.1, and the consensus tree was obtained. The first 25% trees were discarded as burn-in. The ML tree was inferred based on dataset PCGAA under the heterogeneous model LG + C60 + F using IQ-TREE 1.6.10 and 1000 ultrafast bootstraps [44]. Finally, the phylogenetic trees were visualized in FigTree 1.4.4 [45].

### 2.4. Divergence Time Estimation

The divergence time was estimated under the uncorrelated lognormal relaxed clock using MCMCtree in BEAST v.2.5.0 [46] with 1,000,000 generations, with “independent rates” as clock, “300 Ma” as root age, “JC69” as model and default for other parameters. The topology obtained in phylogenetic analysis above was constrained using the following four minimum age calibrations (hard bounds): (1) the most recent common ancestor (MRCA) of Hemerobiidae and Chrysopidae was calibrated with the fossil evidence of *Mesypochrysa minuta* to a minimum age of 165 Ma [47]; (2) the divergence of Apochrysinae from Nothochrysinae was calibrated with a minimum age of 53 Ma based on the fossil evidence of *Adamsochrysa* [48]; (3) the divergence of *Leucochrysa* from *Italochrysa* + *Abachrysa* was calibrated with a minimum age of 21 Ma based on the fossil evidence of *Leucochrysa* (*Nodita*) *prisca* [49]; (4) the divergence of *Chrysoperla* was calibrated with a minimum age of 21 Ma based on the fossil species *Chrysopa glaesaria* [49]. The analysis was terminated until all the effective sample size (ESS) exceeded 200. The first 25% generation was removed as burn-in. Finally, the tree visualization was carried out using FigTree 1.4.4 [45].

## 3. Results

### 3.1. Genome Organization and Base Composition

**Figure 2:**
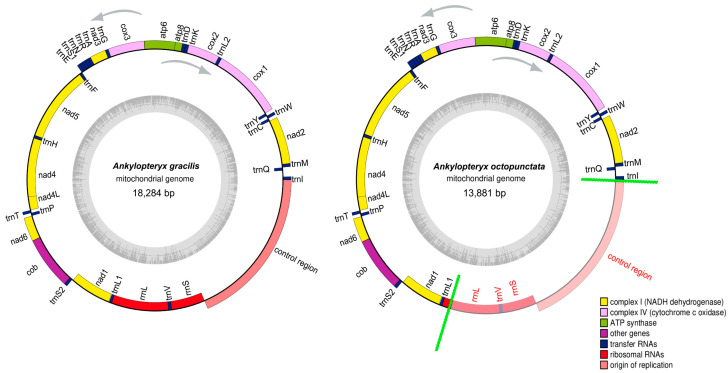
Mitochondrial maps of *Ankylopteryx gracilis* and *Ankylopteryx octopunctata*. The tRNAs, rRNAs and PCGs are denoted by the color blocks. Genes outside the map are transcribed counter-clockwise, whereas those inside are transcribed clockwise. The red font between the green lines in *Ankylopteryx octopunctata* represents the missing region.

The complete mitogenome of *A. gracilis* was 18,284 bp in length, while that of *A. octopunctata* is incomplete with a length of 13,881 bp. The mitogenome sequence of *A. octopunctata* was not complete since the high AT content of the control region (AT-rich region). The complete mitogenome was composed of 13 PCGS, 22 tRNAs, 2 rRNAs and a control region (Figure 2, Tables S2 and S3) including twenty-three genes (9 PCGs, 14 tRNAs) transcribed on the heavy strand (J-strand) and the other fourteen genes (4 PCGs, 8 tRNAs and 2 rRNAs) oriented on the light strand (N-strand).

**Figure 3:**
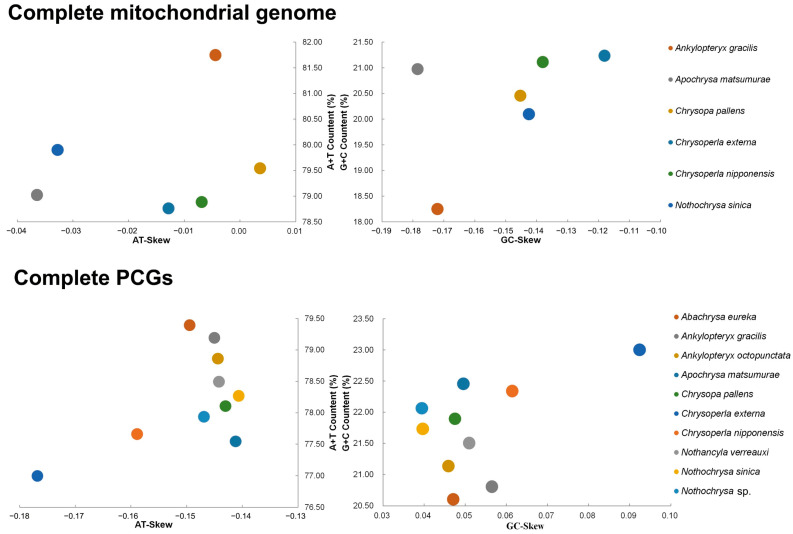
AT% vs. AT-Skew and GC% vs. GC-Skew in complete mitogenomes and PCGs of Chrysopidae.

In total, there are six complete mitogenomes of Chrysopidae including mitogenomes published on GenBank, ranging from 16,057 bp to 18,284 bp. The shortest one was *Chrysoperla nipponensis* (16,057 bp) and the longest was *An. gracilis* (18,284 bp). In the mitogenomes of Chrysopidae, the content of C was higher than G and the content of A was higher than T except *C. pallens*. The A + T content was 78.76% to 81.75% (Figure 3, Table S4). The length of complete PCGs in the available Chrysopidae mitogenomes ranges from 11,127 bp to 11,151 bp. The shortest one was that of *An. octopunctata* (11,127 bp) and the longest were those of *Ch. externa* and *Ch. nipponensis* (11,151 bp). The highest AT content was observed in *Abachrysa eureka* (79.39%) and the lowest AT content was detected in *Ch. externa* (77.0%) (Figure 3, Table S5).

**Figure 4:**
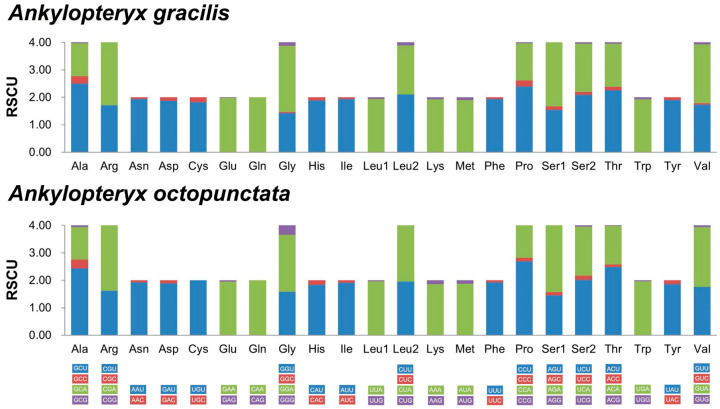
Relative synonymous codon usages (RSCUs) of *Ankylopteryx gracilis* and *Ankylopteryx octopunctata*.

In the complete mitogenomes of Chrysopidae, the highest AT-skew was in *C. pallens* (0.00), and the weakest AT-skew was in *Apochrysa matsumurae* (−0.04). The highest GC-skew was in *Ch. externa* (−0.12), and the weakest GC-skew was in *Ap. matsumurae* (−0.18) (Figure 3, Table S4). In the complete PCGs of mitogenomes of Chrysopidae, the strongest AT-skew was in *Nothancyla sinica* (−0.14) and the weakest AT-skew was in *Ch. externa* (−0.18). The strongest GC-skew was in *Ch. externa* (0.09), and the weakest GC-skew was in *Nothochrysa* sp. (0.04) (Figure 3, Table S5). Compared to the base content of other species, *Ch. externa* was special. It shows a lower content of base A and a higher content of base G (Table S5). Analysis of the base composition at three sites of PCGs revealed a variation in the presence of bases G and C at the third site. Interestingly, our analysis discovered that despite the differences in the third base, the encoded amino acids remained consistent (Tables S6 and S7). This phenomenon was attributed to the degeneracy of codons [50]. The statistical analysis of amino acids encoded by different codons are shown in Figure 4.

### 3.2. Protein-Coding Genes and Codon Usage

Start codons and stop codons of most PCGs were identified except the incomplete *nad2* of *Italochrysa insignis*, *L. pretiosa* and *Parankylopteryx* sp. The use of start codons in Chrysopidae was a typical form with ATN in all PCGs (Table S8). Most PCGs (*atp6*, *atp8*, *cox3*, *nad1* and *nad4L*) used TAA as stop codons in all species, while other PCGs used TAA/TAG or TA-tRNA/T-tRNA as stop codons. The stop codons of *nad3* were TAA/TAG or T-tRNA in different species, and *nad5* was terminated with T-tRNA in all species. The stop codons of *cox1*, *cox2* and *nad4* were T-tRNA in most species, and the stop codons of *cob*, *nad2* and *nad6* were TAA in most species.

The relative synonymous codon usages (RSCUs) of PCGs in the two newly sequenced mitogenomes were shown in Figure 4 (Tables S9 and S10). The codons CGG (Arg), CGC (Arg), CUC (Leu2) and AGG (Thr) were not used in *An. gracilis* and *An. octopunctata*. The most common codons were UUA (Leu1), AUU (Ile), UUU (Phe) and AUA (Met), indicating the preference of nucleotide composition for A/T.

**Figure 5:**
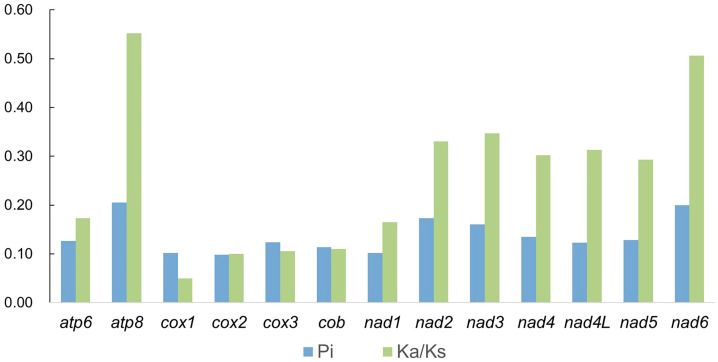
The nucleotide diversity (Pi) and non-synonymous (Ka) to synonymous (Ks) substitution rates of 13 PCGs among Chrysopidae.

The nucleotide diversity (Pi) of 13 PCGs among Chrysopidae was shown in Figure 5. The Pi ranged from 0.098 (*cox2*) to 0.205 (*atp8*). The gene *atp8* (Pi = 0.205) showed the highest nucleotide diversity. There was also relatively high nucleotide diversity in *nad6* (Pi = 0.200) and *nad2* (Pi = 0.173), while *cox2* (Pi = 0.098) and *cox1* (Pi = 0.102) showed relatively low nucleotide diversity, which suggested they were conserved genes. In order to better understand the evolution of PCGs, the rate of nucleotide substitution (Ka/Ks) of PCGs among Chrysopidae was analyzed (Figure 5). The Ka/Ks of 13 PCGs were lower than 1, meaning that Chrysopidae were under purifying selection [51]. The Ka/Ks ranged from 0.049 (*cox1*) to 0.552 (*atp8*), indicating that *cox1* had the slowest evolution rate, while *atp8* had the fastest evolving rate.

### 3.3. The Control Region and Overlapping Regions

**Figure 6:**
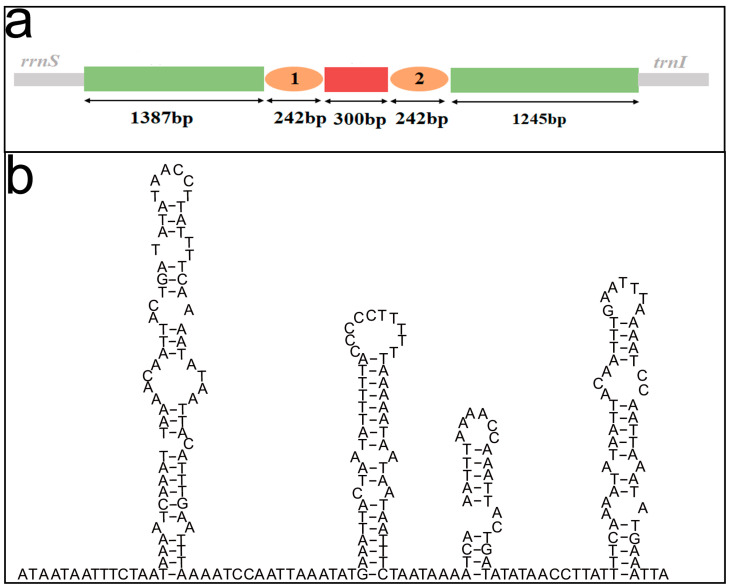
(**a**) The structure of the control region of *Ankylopteryx gracilis*. The two orange sections represent two long repeats of more than 200 bp. (**b**) The possible secondary structures within the two long repeats of the control region in the mitogenome of *An. gracilis*.

The control region regulated the replication and transcription of the mtDNA [52,53]. It can be identified by a number of features, such as, (i) unassigned region (UR) (ii) secondary structure with T-rich loops, (iii) high A-T content and (iv) repetitive elements [54,55]. The control region of *An. gracilis* between *rrnS* and *trnI* (3416 bp) is much longer than the other URs (1–136 bp). Some predicted secondary structures contain T-rich loops (Figure 6). The A-T content (89.38%) is much higher than that of the whole mitogenomes (81.75%). Many short repeats were also found in the control region of *An. gracilis* (Table S11). In the whole mitogenomes of Chrysopidae, the length of the control region was 1244–3416 bp (Table S12). The content of A was higher than T except *Ap. matsumurae* and *Nothancyla sinica*, and the content of G was higher than C. Many microsatellite-like sequences were found in the control region of six complete mitogenomes (*An. gracilis*, *Ap. matsumurae*, *Ch. nipponensis*, *C. pallens*, *Ch. externa* and *Nothancyla sinica*) (Table S11). In addition, two long repeats of more than 200 bp were found in the control region of *An. gracilis* and the predicted secondary structures are shown in Figure 6.

There were overlapping regions of seven nucleotides between the gene pairs *atp8*-*atp6* and *nad4*-*nad4L*, which had been reported in many other insect mitogenomes [56]. All the 13 species shared the same overlapped sequence ATGTTAA between *nad4*-*nad4L*, while the sequences were ATGATAA between *atp8*-*atp6* in most Chrysopiadae and ATGGTAA in *Ch. externa*. In addition, there was an overlapped nucleotide ‘A’ between *atp6* and *cox3* found in the 13 Chrysopidae species.

### 3.4. Transfer RNAs and Ribosomal RNAs

**Figure 7:**
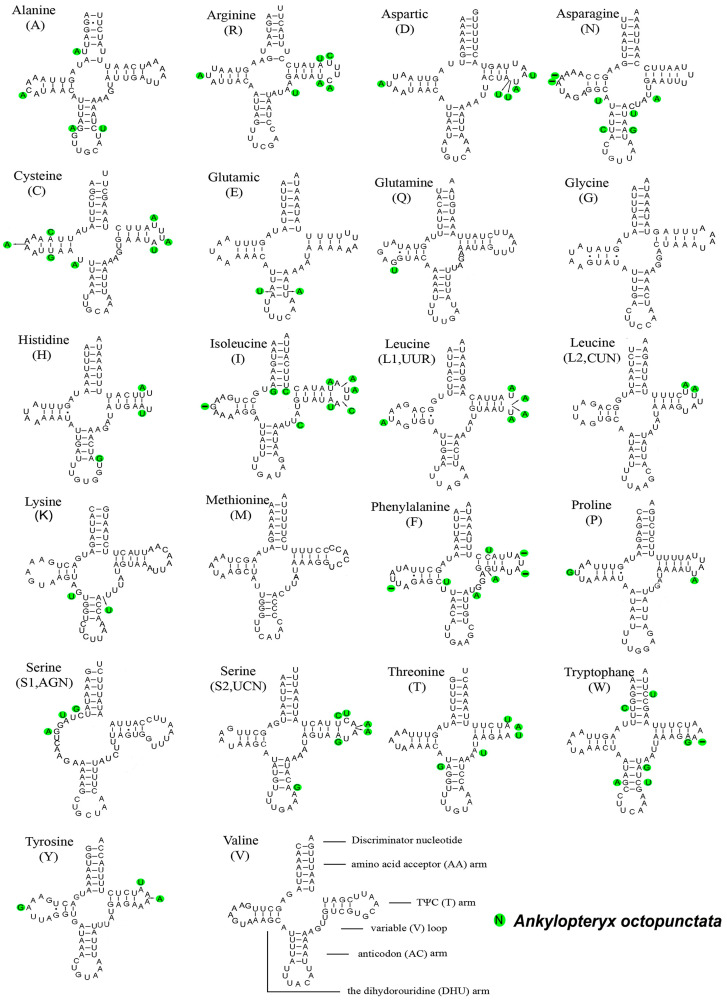
Predicted secondary structures of 22 tRNAs in *Ankylopteryx gracilis* and comparison with that of *An. octopunctata*. The green circles represent the bases that differ between the two species.

The secondary structures of tRNAs of *An. gracilis* were predicted and compared with those of *An. octopunctata* (Figure 7). There was little variation in all the 22 tRNAs. The *trnG* and *trnM* were identical. The greatest difference was in *trnI* with nine sites changed. Almost all tRNAs could be folded into cloverleaf structures, except for *trnS2*, whose dihydrouridine (DHU) arm formed a simple loop. This characteristic occurred frequently in the sequenced mitogenomes of metazoan [57].

**Figure 8:**
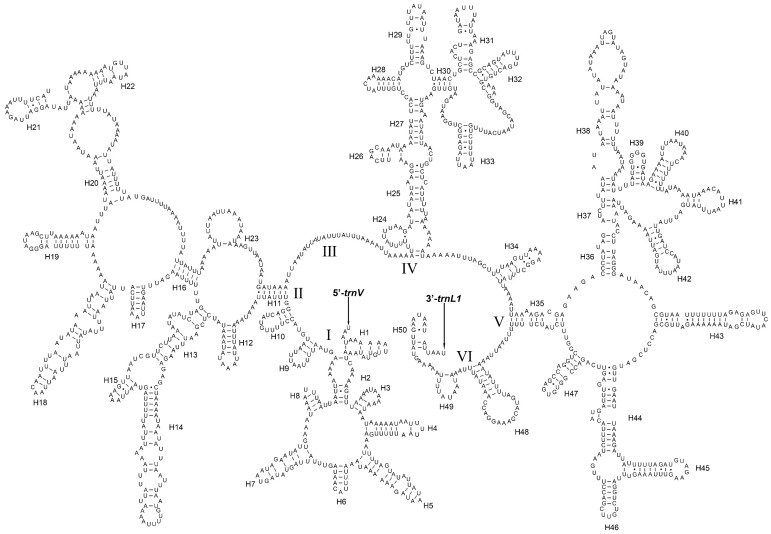
Predicted secondary structure of *rrnL* in *Ankylopteryx gracilis*. The I–VI represents six domains

The *rrnL* was located between *trnL1* and *trnV*. The length of *rrnL* was 1301–1319 bp. The A + T content ranged from 81.64% to 83.85% (Table S13). The content of A was higher than T except *An. gracilis*, *C. pallens* and *Nothancyla verreauxi*, and the content of G was higher than C. The secondary structure of *rrnL* of *An. gracilis* was predicted (Figure 8). There were five domains (I, II, IV, V and VI) and 50 helices in the secondary structure of *rrnL*. The domain III deletion of *rrnL* is a typical feature of arthropods [58].

**Figure 9:**
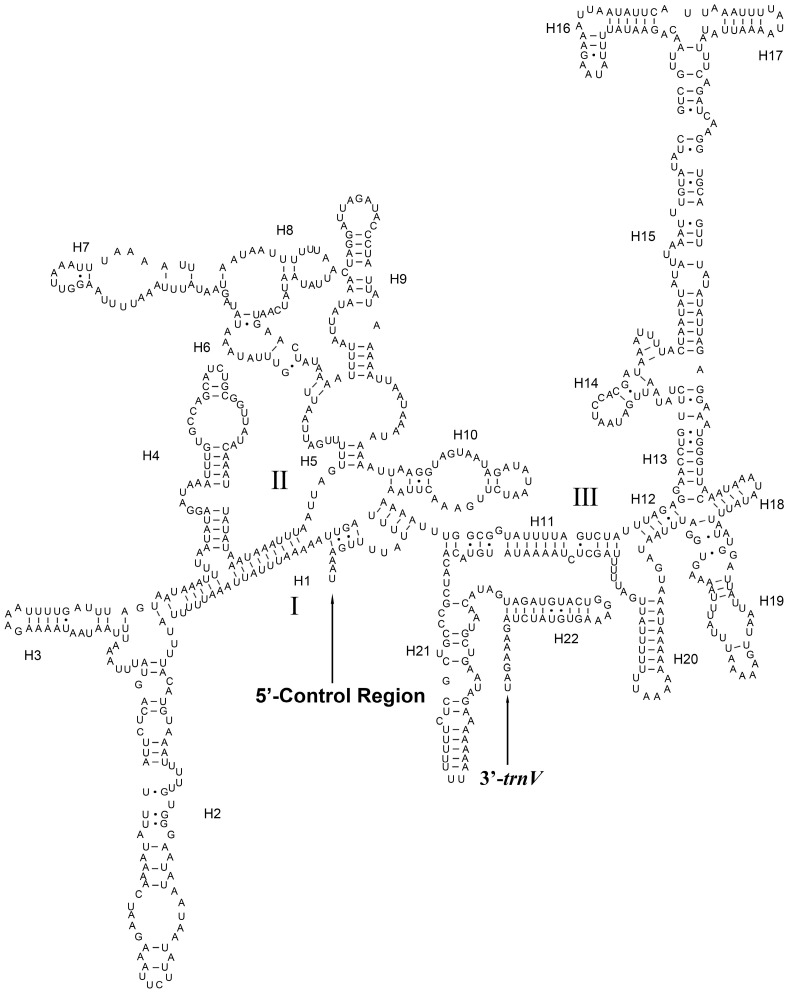
Predicted secondary structure of *rrnS* in *Ankylopteryx gracilis*. The I–III represents three domains

The *rrnS* was located between *trnV* and the control region. The length of *rrnS* was 773–784 bp (Table S13). The A + T content ranged from 80.15% to 82.32%. The content of A was higher than T except *Ab. eureka* and *C. pallens,* and the content of G was higher than C. The predicted secondary structure of *rrnS* of *An. gracilis* was shown in Figure 9. There were three domains and 22 helices in the secondary structure of *rrnS*.

### 3.5. Phylogenetic Analyses

**Figure 10:**
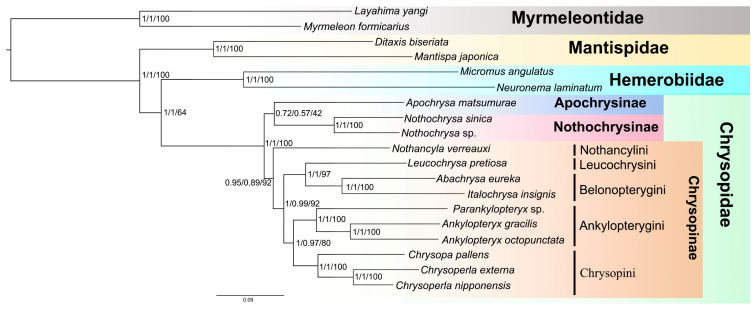
Phylogeny inferred from Bayesian analysis based on the dataset PCG123RNA and PCGAA under the heterogeneous model CAT-GTR. Branch support values are featured at respective node in the following order: posterior probabilities (based on PCG123RNA under heterogeneous CAT-GTR model)/posterior probabilities (based on PCGAA under heterogeneous CAT-GTR model)/bootstrap values (based on PCGAA under heterogeneous LG + C60 + F model).

The phylogenetic trees were reconstructed under the heterogeneous models CAT + GTR (datasets PCG123RNA and PCGAA) and LG + C60 + F (dataset PCGAA), respectively (Figure 10). The topologies were consistent in these three analyses. Chrysopinae were recovered as the sister to Apochrysinae + Nothochrysinae. However, the sister group relationship between Apochrysinae and Nothochrysinae was at low support. Nothancylini were recovered as the sister group to other Chrysopinae. Leucochrysini were recovered as the sister group to Belonopterygini, while Ankylopterygini were recovered as the sister group to Chrysopini with high support.

### 3.6. Divergence Time Estimation

The divergence time was estimated based on the phylogeny of dataset PCG123RNA and the chronogram was shown in Figure 9. Mean age values and 95% high posterior density (HPD) intervals for each node are presented in Table 1. The initial divergence with extant Chrysopidae was estimated occurring in the Early Cretaceous (~135 Ma; 95% HPD = 103.36–174.95 Ma). Apochrysinae diverged from Nothochrysinae slightly later during the Early Cretaceous (~115 Ma; 95% HPD = 86.37–150.55 Ma). Within Chrysopinae, Nothancylini diverged from other tribes during the Early Cretaceous (~125 Ma; 95% HPD = 95.39–161.98 Ma). Leucochrysini + Belonopterygini diverged from Ankylopterygini + Chrysopini also during the Early Cretaceous (~113 Ma; 95% HPD = 86.24–147.39 Ma). Leucochrysini diverged from Belonopterygini during the Late Cretaceous (~93 Ma; 95% HPD = 69.23–122.79 Ma). Ankylopterygini diverged from Chrysopini during the Middle Cretaceous (~101 Ma; 95% HPD = 76.57–132.09 Ma). Within Ankylopterygini, *Parankylopteryx* diverged from *Ankylopteryx* during the Late Cretaceous (~83 Ma; 95% HPD = 60.85–108.46 Ma). The divergence between *An. gracilis* and *An. octopunctata* occurred during the early Paleogene (~59 Ma; 95% HPD = 41.81–79.46 Ma).

## 4. Discussion and Conclusions

The mitogenomes of *An. gracilis* and *An. octopunctata* were newly sequenced and analyzed in this study. The arrangements of these two mitogenomes were consistent with other Chrysopidae species. The use of start codons of Chrysopidae was relatively simple with the form of ATN. Most PCGs used TAA as stop codons, while some PCGs used TA-tRNA or T-tRNA as stop codons. Incomplete stop codons TA-tRNA and T-tRNA would transform into complete stop codons after transcription in Chrysopidae [50].

Concerning the phylogeny of Chrysopidae, Chrysopinae were recovered as the sister group to Apochrysinae + Nothochrysinae. This result had also been confirmed by many studies using molecular evidence, such as nuclear genes, mitogenomes and low-coverage whole genomes [10,11,12,13,16]. Apochrysinae were recovered as the sister group to Nothochrysinae with low support. Traditional morphological studies considered Nothochrysinae as the sister group to the rest of the Chrysopidae based on fossil records and plesiomorphic similarities [2,6,7,14,17,59,60] without any molecular evidence supporting it. Mitogenomes provide more genetic information compared to single genes, which can better reflect phylogenetic relationships [61,62]. However, there is still a significant gap comparing with low-coverage whole genomes and AHE data [15,16]. The combination of molecular and morphological evidence provides a more comprehensive explanation of phylogeny. Additionally, increasing the number of samples in studies can lead to more reliable results. In a recent study, Breitkreuz et al. recovered Apochrysinae as the sister group to Nothochrysinae + Chrysopinae using a large number of samples based on morphological and molecular data [17]. This conclusion has been endorsed by many researchers. Within Chrysopinae, Nothancylini were recovered as the sister group to (Leucochrysini + Belonopterygini) + (Chrysopini + Ankylopterygini), which is consistent with previous studies using mitogenomes from studies of Jiang et al. and Zhang et al. [12,13]. The three extant subfamilies of Chrysopidae diverged from each other during the Early Cretaceous, which is consistent with previous results based on molecular data [14,15]. However, the divergence times among the chrysopine tribes were estimated as earlier than previous results [14,15]. In order to better resolve the phylogenetic relationships and divergence time estimation among Chrysopidae, much more mitogenomes and molecular data in other forms are needed in the future.

## Figures and Tables

**Figure 11:**
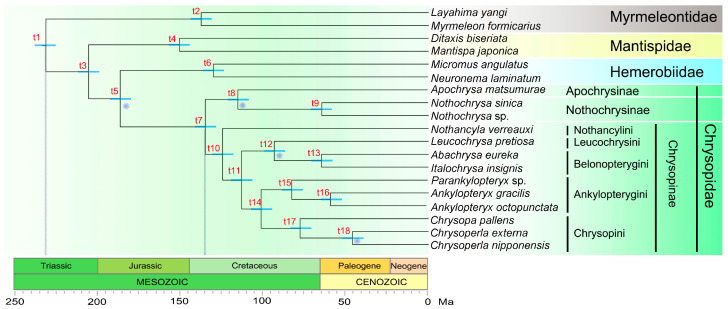
The divergence time estimations calculated using fossil calibration (starts at nodes) are featured. The age estimated for each node are presented in Table 1. The grey symbol represents age calibration from fossil evidence.

**Table 1:** Mean divergence times and 95% high posterior density (HPD) intervals for each node of the topology presented in Figure 11.

Node	Mean	Inferior 95%	Superior 95%	ESS	Crown Clade
t1	231.51	179.28	297.26	425.30	
t2	137.62	89.43	189.82	759.50	Myrmeleontidae
t3	205.66	162.42	263.75	378.50	
t4	150.78	108.51	200.38	516.20	Mantispidae
t5	186.55	148.50	240.02	378.50	Hemerobiidae + Chrysopidae
t6	130.15	93.80	173.69	518.10	Hemerobiidae
t7	135.09	103.36	174.95	360.40	Chrysopidae
t8	115.36	86.37	150.55	388.80	Apochrysinae + Nothochrysinae
t9	64.56	42.95	88.37	620.90	Nothochrysinae
t10	124.52	95.39	161.98	360.10	Chrysopinae
t11	113.04	86.24	147.39	361.30	
t12	93.33	69.23	122.79	396.30	Leucochrysini + Belonopterygini
t13	64.83	44.96	87.14	488.50	Belonopterygini
t14	101.24	76.57	132.09	368.30	Ankylopterygini + Chrysopini
t15	82.71	60.85	108.46	398.50	Ankylopterygini
t16	59.31	41.81	79.46	480.90	Ankylopterygini
t17	77.77	56.76	102.66	418.60	Chrysopini
t18	46.08	31.14	62.89	573.30	Chrysopini

## Data Availability

The data supporting the findings of this study are openly available from the National Center for Biotechnology Information at https://www.ncbi.nlm.nih.gov (accessed on 18 January 2023), accession numbers: OQ269716 and OM510943.

## Supplementary Materials

The following supporting information can be downloaded at: https://www.mdpi.com/article/10.3390/insects14110878/s1. Table S1: List of taxonomic groups used for the phylogenetic analyses in this study [12,13,30,31,32,33,34,35,36]; Table S2: Organization of *Ankylopteryx gracilis* mitogenome; Table S3: Organization of *Ankylopteryx octopunctata* mitogenome; Table S4: Nucleotide composition and skews in the complete mitogenomes of Chrysopidae; Table S5: Nucleotide composition and skews in the complete PCGs of Chrysopidae; Table S6: Nucleotide composition at the three codon sites in the complete PCGs of Chrysopidae. Table S7: Encoded amino acids composition in the complete PCGs of Chrysopidae. Table S8: Start codons and Stop codons of PCGs in Chrysopidae; Table S9: Relative synonymous codon usages (RSCUs) of PCGs of *Ankylopteryx gracilis*; Table S10: Relative synonymous codon usages (RSCUs) of PCGs of *Ankylopteryx octopunctata*; Table S11: The count of microsatellite-like sequence in the complete control regions of Chrysopidae; Table S12: Nucleotide composition and skews in the complete control regions of Chrysopidae; Table S13: Nucleotide composition and skews in the rRNAs.
