# Supplementary material for: New Mitogenomes of the Green Lacewing Tribe Ankylopterygini (Neuroptera: Chrysopidae: Chrysopinae) and Phylogenetic Implications of Chrysopidae
Source: Insects. 2023 Nov 14;14(11):878. doi: 10.3390/insects14110878 (PMC10672194; doi:10.3390/insects14110878)
Supplement: Supplementary file 1 [file insects-14-00878-s001.zip › insects-2658232-supplementary.pdf]

Table S1. List of taxonomic groups used for the phylogenetic analyses in this study.

Family	Species	Accession number	Reference
Chrysopidae	*Abachrysa eureka*	KY587199	[13]
	Ankylopteryx gracilis	OQ269716	This study
	Ankylopteryx octopunctata	OM510943	This study
	Apochrysa matsumurae	NC_015095	[30]
	Chrysopa pallens	NC_019618	[31]
	Chrysoperla externa	NC_030341	
	Chrysoperla nipponensis	NC_015093	[30]
	Italochrysa insignis	KY587200	[13]
	Leucochrysa pretiosa	KY587201	[13]
	Nothancyla verreauxi	KP264629	[13]
	Nothochrysa sinica	NC_060341	[12]
	Nothochrysa sp.	KP264630	
	Parankylopteryx sp.	KY587202	[13]
Hemerobiidae	*Neuronema laminatum*	NC_028153	[32]
	Micromus angulatus	NC_042677	[33]
Mantispidae	*Ditaxis biseriata*	NC_013257	[34]
	Mantispa japonica	NC_039771	[35]
Myrmeleontidae	*Layahima yangi*	NC_071191	
	Myrmeleon formicarius	NC_047285	[36]

References

1. Jiang, Y.; Garzón-Orduña, I.J.; Winterton, S.L.; Yang, F.; Liu, X. Phylogenetic relationships among tribes of the green lacewing subfamily Chrysopinae recovered based on mitochondrial phylogenomics. *Sci. Rep.* **2017**, *7*, 7218. https://doi.org/10.1038/s41598-017-07431-1.
2. Haruyama, N.; Mochizuki, A.; Sato, Y.; Naka, H.; Nomura, M. Complete mitochondrial genomes of two green lacewings, *Chrysoperla nipponensis* (Okamoto, 1914) and Apochrysa matsumurae Okamoto, 1912 (Neuroptera: Chrysopidae). *Mol. Biol. Rep.* **2011**, *38*, 3367-3373, doi:10.1007/s11033-010-0444-0.
3. He, K.; Chen, Z.; Yu, D.N.; Zhang, J.Y. The complete mitochondrial genome of *Chrysopa pallens* (Insecta, Neuroptera, Chrysopidae). *Mitochondrial DNA* **2012**, *23*, 373-375, doi:10.3109/19401736.2012.696631.
4. Zhang, R.Y.; Ma, Y.L.; Fan, F.; Geng, S.; Wang, Y.Y.; Liu, X.Y. The complete mitochondrial genome of *Nothochrysa sinica* (Neuroptera: Chrysopidae: Nothochrysinae) with a phylogenetic analysis of Chrysopoidea. *Mitochondrial DNA Part B* **2021**, *6*, 1632–1633. https://doi.org/10.1080/23802359.2021.1926361.
5. Zhao, Y.; Chen, Y.J.; Zhao, J.; Liu, Z.Q. First complete mitochondrial genome from the brown lacewings (Neuroptera: Hemerobiidae). *Mitochondrial DNA Part A* **2016**, *27*, 2763-2764, doi:10.3109/19401736.2015.1053054.
6. Park, J.; Park, J.; Kim, J.H.; Cho, J.R.; Kim, Y.; Seo, B.Y. The complete mitochondrial genome of *Micromus angulatus* (Stephens, 1836) (Neuroptera: Hemerobiidae). *Mitochondrial DNA Part B* **2019**, *4*, 1467-1469, doi:10.1080/23802359.2019.1598800.
7. Cameron, S.L.; Sullivan, J.; Song, H.; Miller, K.B.; Whiting, M.F. A mitochondrial genome phylogeny of the Neuropterida (lace-wings, alderflies and snakeflies) and their relationship to the other holometabolous insect orders. *Zool. Scr.* **2009**, *38*, 575-590, doi:10.1111/j.1463-6409.2009.00392.x.

8. Chen, T.; Lan, X.E.; Chen, X.N.; You, P. Studies on complete mitochondrial genomes and Neuroptera phylogenesis of three Mantispidae. *Journal of Shaanxi Normal University* **2017**, *46*, 81-92.
9. Wu, J.Q.; Wang, S.Z.; Yang, X.Z.; Guo, Y.J.; Weng, X.Q.; Wu, S.Q. The complete mitochondrial genome of *Myrmeleon formicarius* (Neuroptera: Myrmeleontidae). *Mitochondrial DNA Part B* **2020**, *5*, 1798-1799, doi:10.1080/23802359.2020.1750977.

Table S2. Organization of *Ankylopteryx gracilis* mitogenome.

Gene	Direction	Location		Size	Anticodon	Codon		Intergenic
		Start	End			Start	Stop	nucleotides
trnI	J	1	69	69	GAT			136
trnQ	N	274	206	69	TTG			2
trnM	J	277	344	68	CAT			0
nad2	J	345	1353	1009		ATT	T-tRNA	0
trnC	N	1416	1354	63	GCA			3
trnW	J	1420	1484	65	TCA			1
trnY	N	1549	1486	64	GTA			3
cox1	J	1553	3086	1534		TCG	T-tRNA	0
trnL2	J	3087	3150	64	TAA			4
cox2	J	3155	3838	684		ATG	TAA	2
trnK	J	3841	3910	70	CTT			0
trnD	J	3911	3975	65	GTC			0
atp8	J	3976	4134	159		ATT	TAA	-7
atp6	J	4128	4805	678		ATG	TAA	-1
cox3	J	4805	5593	789		ATG	TAA	2
*trn*G	J	5596	5660	65	TCC			0
nad3	J	5661	6012	352		ATT	T-tRNA	0
trnA	J	6013	6078	66	TGC			0
trnR	J	6079	6140	62	TCG			0
trnN	J	6141	6208	68	GTT			0
trnS1	J	6209	6275	67	TTC			1

trnE	J	6277	6340	64	TTC			-2
trnF	N	6404	6339	66	GAA			0
nad5	N	8130	6405	1726		ATT	T-tRNA	0
trnH	N	8193	8131	63	GTG			0
nad4	N	9530	8194	1337		ATG	TA-tRNA	-7
nad4L	N	9820	9524	297		ATG	TAA	2
trnT	J	9823	9886	64	TGT			0
trnP	N	9952	9887	66	TGG			2
nad6	J	9955	10464	510		ATT	TAA	-1
cob	J	10464	11598	1135		ATG	T-tRNA	0
trnS2	J	11599	11665	67	TGA			24
nad1	N	12628	11690	939		ATA	TAA	13
trnL1	N	12704	12642	63	TAG			0
rrnL	N	14023	12705	1319				0
trnV	N	14093	14024	70	TAC			0
rrnS	N	14868	14094	775				0
Control region		14869	18284	3416				

Table S3. Organization of *Ankylopteryx octopunctata* mitogenome.

Gene	Direction	Location		Size	Anticodon	Codon		Intergenic
		Start	End			Start	Stop	nucleotides
trnI	J	328	394	67	GAT			137
trnQ	N	600	532	69	TTG			2
trnM	J	603	670	68	CAT			0
nad2	J	671	1679	1009		ATT	T-tRNA	0
trnC	N	1743	1680	64	GCA			1
trnW	J	1745	1808	64	TCA			1
trnY	N	1874	1810	65	GTA			2
cox1	J	1877	3410	1534		TCG	T-tRNA	0
trnL2	J	3411	3474	64	TAA			3
cox2	J	3478	4159	682		ATG	T-tRNA	0
trnK	J	4160	4230	71	CTT			-1
trnD	J	4230	4295	66	GTC			0
atp8	J	4296	4454	159		ATC	TAA	-7
atp6	J	4448	5125	678		ATG	TAA	-1
cox3	J	5125	5913	789		ATG	TAG	3
*trn*G	J	5917	5981	65	TCC			0
nad3	J	5982	6333	352		ATT	T-tRNA	0
trnA	J	6334	6399	66	TGC			-1
trnR	J	6399	6462	64	TCG			3
trnN	J	6466	6531	66	GTT			0
trnS1	J	6532	6598	67	TTC			8

trnE	J	6607	6671	65	TTC			-2
trnF	N	6732	6670	63	GAA			0
nad5	N	8458	6733	1726		ATT	T-tRNA	0
trnH	N	8521	8459	63	GTG			0
nad4	N	9855	8522	1334		ATG	TA-tRNA	-7
nad4L	N	10145	9849	297		ATG	TAA	2
trnT	J	10148	10211	64	TGT			0
trnP	N	10277	10212	66	TGG			1
nad6	J	10279	10788	510		ATT	TAA	-1
cob	J	10788	11922	1135		ATG	T-tRNA	0
trnS2	J	11923	11989	67	TGA			30
nad1	N	12949	12020	930		ATA	TAA	22
trnL1	N	13034	12972	63	TAG			

Table S4. Nucleotide composition and skews in the complete mitogenomes of Chrysopidae.

Species	T(U)	C	A	G	Total	A+T%	AT-Skew	GC-Skew
Ankylopteryx gracilis	41.05	10.70	40.69	7.56	18284.0	81.75	0.00	-0.17
Apochrysa matsumurae	40.95	12.36	38.07	8.62	16214.0	79.02	-0.04	-0.18
Chrysopa pallens	39.63	11.71	39.92	8.74	16723.0	79.54	0.00	-0.15
Chrysoperla externa	39.89	11.87	38.88	9.36	16509.0	78.76	-0.01	-0.12
Chrysoperla nipponensis	39.71	12.01	39.17	9.10	16057.0	78.89	-0.01	-0.14
Nothochrysa sinica	41.26	11.48	38.64	8.62	16166.0	79.90	-0.03	-0.14

Table S5. Nucleotide composition and skews in the complete PCGs of Chrysopidae.

Species	T(U)	C	A	G	Total	A+T%	AT-Skew	GC-Skew
Abachrysa eureka	45.6	9.8	33.8	10.8	11133.0	79.39	-0.15	0.05
Ankylopteryx gracilis	45.3	9.8	33.9	11.0	11145.0	79.19	-0.15	0.06
Ankylopteryx octopunctata	45.1	10.1	33.7	11.1	11127.0	78.86	-0.14	0.05
Apochrysa matsumurae	44.2	10.7	33.3	11.8	11142.0	77.54	-0.14	0.05
Chrysopa pallens	44.6	10.4	33.5	11.5	11154.0	78.11	-0.14	0.05
Chrysoperla externa	45.3	10.4	31.7	12.6	11151.0	77.00	-0.18	0.09
Chrysoperla nipponensis	45.0	10.5	32.7	11.9	11151.0	77.66	-0.16	0.06
Nothancyla verreauxi	44.9	10.2	33.6	11.3	11132.0	78.49	-0.14	0.05
Nothochrysa sinica	44.6	10.4	33.6	11.3	11136.0	78.27	-0.14	0.04
Nothochrysa sp.	44.7	10.6	33.2	11.5	11145.0	77.94	-0.15	0.04

Table S6. Nucleotide composition at the three codon sites in the complete PCGs of Chrysopidae.

Species	T-1	C-1	A-1	G-1	T-2	C-2	A-2	G-2	T-3	C-3	A-3	G-3
Abachrysa eureka	39.0	9.7	34.2	17.3	47.0	17.8	21.5	14.1	52.0	2.0	45.5	1.0
Ankylopteryx gracilis	38.0	9.7	34.5	17.6	47.0	17.4	21.8	13.9	51.0	2.3	45.3	1.5
Ankylopteryx octopunctata	38.0	9.9	34.9	17.4	47.0	17.8	21.6	14.2	51.0	2.5	44.8	1.6
Apochrysa matsumurae	38.0	10.6	33.3	18.3	46.0	18.4	21.1	14.2	49.0	3.0	45.4	2.8
Chrysopa pallens	38.0	10.1	33.2	18.4	46.0	18.2	21.3	14.2	49.0	3.0	46.0	1.9
Chrysoperla externa	39.0	10.0	32.5	18.9	46.0	18.6	21.3	14.2	51.0	2.8	41.3	4.6
Chrysoperla nipponensis	39.0	9.9	32.2	19.3	46.0	18.7	21.0	14.4	51.0	2.8	44.7	1.9
Nothancyla verreauxi	38.0	9.9	34.1	18.1	46.0	18.3	21.5	14.1	51.0	2.4	45.2	1.7
Nothochrysa sinica	38.0	10.3	33.9	18.0	46.0	18.5	21.5	14.1	50.0	2.5	45.5	1.8
Nothochrysa sp.	37.0	10.5	33.8	18.3	46.0	18.7	21.3	14.3	51.0	2.6	44.6	1.9

Table S7. Encoded amino acids composition in the complete PCGs of Chrysopidae.

Species	Ala	Cys	Asp	Glu	Phe	Gly	His	Ile	Lys	Leu	Met	Asn	Pro	Gln	Arg	Ser	Thr	Val	Trp	Tyr
Abachrysa eureka	4.11	1.00	1.78	2.05	8.89	5.46	1.97	10.86	2.35	16.10	6.94	6.19	3.43	1.89	1.54	9.00	4.65	3.94	2.76	5.11
Ankylopteryx gracilis	4.02	0.92	1.67	2.10	8.76	5.50	2.00	11.00	2.32	15.97	6.93	6.47	3.43	1.81	1.51	8.79	4.53	4.29	2.72	5.26
Ankylopteryx octopunctata	4.08	1.05	1.84	2.00	8.64	5.48	2.00	10.99	2.38	15.74	7.16	6.10	3.38	1.84	1.54	8.91	4.86	4.05	2.67	5.29
Apochrysa matsumurae	4.43	0.97	1.78	2.05	9.12	5.51	2.08	10.26	2.51	15.76	6.61	5.59	3.43	1.92	1.57	9.20	4.91	4.59	2.70	5.02
Chrysopa pallens	4.34	1.00	1.91	2.08	8.95	5.55	2.00	10.51	2.37	16.10	6.39	5.88	3.50	1.78	1.54	9.09	4.69	4.53	2.72	5.07
Chrysoperla externa	4.53	0.97	1.89	2.10	8.79	5.72	1.97	9.98	2.27	16.40	6.18	5.88	3.45	1.83	1.54	8.90	5.02	4.69	2.72	5.18
Chrysoperla nipponensis	4.53	0.97	1.97	2.10	8.76	5.72	2.00	9.76	2.21	16.26	6.18	5.66	3.48	1.86	1.54	9.09	5.07	5.04	2.75	5.04
Nothancyla verreauxi	4.43	1.03	1.84	2.05	8.65	5.59	1.97	10.38	2.43	15.89	7.08	6.05	3.46	1.81	1.49	8.78	4.94	4.24	2.73	5.16
Nothochrysa sinica	4.32	0.97	1.84	2.13	8.51	5.59	1.94	10.80	2.46	15.91	6.62	5.97	3.54	1.81	1.54	9.07	4.89	4.21	2.73	5.16
Nothochrysa sp.	4.35	0.94	1.86	2.11	8.53	5.59	1.94	10.53	2.48	15.90	6.45	5.72	3.54	1.84	1.54	9.23	5.16	4.40	2.73	5.18

Table S8. Start codons and stop codons of PCGs in Chrysopidae.

	ATP6	*ATP8*	*COX1*	*COX2*	*COX3*	*CYTB*	*ND1*	*ND2*	*ND3*	*ND4*	*ND4L*	*ND5*	*ND6*
**Start codon**													
Abachrysa eureka	ATG	ATT	TCG	ATG	ATG	ATG	ATA	ATT	ATG	ATG	ATG	ATT	ATT
Ankylopteryx gracilis	ATG	ATT	TCG	ATG	ATG	ATG	ATA	ATT	ATT	ATG	ATG	ATT	ATT
Ankylopteryx octopunctata	ATG	ATC	TCG	ATG	ATG	ATG	ATA	ATT	ATT	ATG	ATG	ATT	ATT
Apochrysa matsumurae	ATG	ATT	TCG	ATG	ATG	ATG	ATA	ATT	ATC	ATG	ATG	ATA	ATT
Chrysopa pallens	ATG	ATT	ATT	ATG	ATG	ATG	ATA	ATT	ATT	ATG	ATG	ATT	ATT
Chrysoperla externa	ATG	ATG	TCG	ATG	ATG	ATG	ATG	ATG	ATG	ATG	ATG	ATG	ATG
Chrysoperla nipponensis	ATG	ATT	TCG	ATG	ATG	ATG	ATG	ATT	ATT	ATG	ATG	ATA	ATT
Italochrysa insignis	ATG	ATT	TCG	ATG	ATG	ATG	ATA		ATT	ATG	ATG	ATT	ATT
Leucochrysa pretiosa	ATG	ATT	TCG	ATG	ATG	ATG	ATA		ATT	ATG	ATG	ATT	ATT
Nothancyla verreauxi	ATG	ATT	TCG	ATG	ATG	ATG	ATA	ATT	ATT	ATG	ATG	ATT	ATT
Nothochrysa sinica	ATG	ATT	TCG	ATG	ATG	ATG	ATT	ATT	ATT	ATG	ATG	ATT	ATT
Nothochrysa sp.	ATG	ATT	TCG	ATG	ATG	ATG	ATT	ATT	ATT	ATG	ATG	ATT	ATT
Parankylopteryx sp.	ATG	ATT	ATT	ATG	ATG	ATG	ATA		ATT	ATG	ATG	ATT	ATT
**Stop codon**													
Abachrysa eureka	TAA	TAA	T-tRNA	T-tRNA	TAA	TAA	TAA	TAA	TAG	T-tRNA	TAA	T-tRNA	TAA
Ankylopteryx gracilis	TAA	TAA	T-tRNA	TAA	TAA	T-tRNA	TAA	T-tRNA	T-tRNA	TA-tRNA	TAA	T-tRNA	TAA
Ankylopteryx octopunctata	TAA	TAA	T-tRNA	T-tRNA	TAA	T-tRNA	TAA	T-tRNA	T-tRNA	TA-tRNA	TAA	T-tRNA	TAA
Apochrysa matsumurae	TAA	TAA	T-tRNA	T-tRNA	TAA	TAA	TAA	TAA	TAG	T-tRNA	TAA	T-tRNA	TAA
Chrysopa pallens	TAA	TAA	T-tRNA	T-tRNA	TAA	TAA	TAA	TAA	TAG	T-tRNA	TAA	T-tRNA	TAA
Chrysoperla externa	TAA	TAA	T-tRNA	T-tRNA	TAA	TAA	TAA	TAA	TAG	TAA	TAA	T-tRNA	T-tRNA
Chrysoperla nipponensis	TAA	TAA	T-tRNA	T-tRNA	TAA	TAA	TAA	TAA	TAG	T-tRNA	TAA	T-tRNA	TAA
Italochrysa insignis	TAA	TAA	T-tRNA	T-tRNA	TAA	TAA	TAA		TAG	T-tRNA	TAA	T-tRNA	TAA
Leucochrysa pretiosa	TAA	TAA	T-tRNA	T-tRNA	TAA	TAA	TAA		TAG	T-tRNA	TAA	T-tRNA	TAA
Nothancyla verreauxi	TAA	TAA	T-tRNA	T-tRNA	TAA	TAA	TAA	TAA	TAG	T-tRNA	TAA	T-tRNA	TAA
Nothochrysa sinica	TAA	TAA	TAA	T-tRNA	TAA	TA-tRNA	TAA	TAA	TAA	T-tRNA	TAA	T-tRNA	TAA
Nothochrysa sp.	TAA	TAA	TAA	T-tRNA	TAA	TAA	TAA	TAA	TAA	T-tRNA	TAA	T-tRNA	TAA
Parankylopteryx sp.	TAA	TAA	T-tRNA	T-tRNA	TAA	TAA	TAA		TAA	T-tRNA	TAA	T-tRNA	TAA

Table S9. Relative synonymous codon usages (RSCUs) of PCGs of *Ankylopteryx gracilis*.

Codon	Count	RSCU	Codon	Count	RSCU
UUU(F)	315	1.94	UCU(S)	107	2.10
UUC(F)	10	0.06	UCC(S)	5	0.10
UUA(L)	539	1.94	UCA(S)	90	1.76
UUG(L)	17	0.06	UCG(S)	2	0.04
UAU(Y)	185	1.90	UGU(C)	31	1.82
UAC(Y)	10	0.10	UGC(C)	3	0.18
UAA(*)	7	2.00	UGA(W)	97	1.92
UAG(*)	0	0.00	UGG(W)	4	0.08
CUU(L)	19	2.11	CCU(P)	76	2.39
CUC(L)	0	0.00	CCC(P)	7	0.22
CUA(L)	16	1.78	CCA(P)	43	1.35
CUG(L)	1	0.11	CCG(P)	1	0.03
CAU(H)	70	1.89	CGU(R)	24	1.71
CAC(H)	4	0.11	CGC(R)	0	0.00
CAA(Q)	65	2.00	CGA(R)	32	2.29
CAG(Q)	2	0.00	CGG(R)	0	0.00
AUU(I)	395	1.94	ACU(T)	95	2.26
AUC(I)	13	0.06	ACC(T)	5	0.12
AUA(M)	244	1.90	ACA(T)	66	1.57
AUG(M)	13	0.10	ACG(T)	2	0.05
AAU(N)	233	1.94	AGU(S)	47	1.54
AAC(N)	7	0.06	AGC(S)	4	0.13
AAA(K)	83	1.93	AGA(S)	71	2.33
AAG(K)	3	0.07	AGG(S)	0	0.00
GUU(V)	69	1.74	GCU(A)	93	2.50
GUC(V)	2	0.05	GCC(A)	10	0.27
GUA(V)	85	2.14	GCA(A)	45	1.21
GUG(V)	3	0.08	GCG(A)	1	0.03
GAU(D)	58	1.87	GGU(G)	73	1.43
GAC(D)	4	0.13	GGC(G)	2	0.04
GAA(E)	77	1.97	GGA(G)	123	2.41
GAG(E)	1	0.03	GGG(G)	6	0.12

*: Stop codon

Table S10. Relative synonymous codon usages (RSCUs) of PCGs of *Ankylopteryx octopunctata*.

Codon	Count	RSCU	Codon	Count	RSCU
UUU(F)	308	1.93	UCU(S)	102	2.01
UUC(F)	12	0.08	UCC(S)	8	0.16
UUA(L)	530	1.97	UCA(S)	91	1.79
UUG(L)	8	0.03	UCG(S)	2	0.04
UAU(Y)	182	1.86	UGU(C)	39	2.00
UAC(Y)	14	0.14	UGC(C)	0	0.00
UAA(*)	5	1.67	UGA(W)	97	1.96
UAG(*)	1	0.00	UGG(W)	2	0.04
CUU(L)	22	1.96	CCU(P)	84	2.69
CUC(L)	0	0.00	CCC(P)	4	0.13
CUA(L)	23	2.04	CCA(P)	37	1.18
CUG(L)	0	0.00	CCG(P)	0	0.00
CAU(H)	68	1.84	CGU(R)	23	1.61
CAC(H)	6	0.16	CGC(R)	0	0.00
CAA(Q)	68	2.00	CGA(R)	34	2.39
CAG(Q)	0	0.00	CGG(R)	0	0.00
AUU(I)	390	1.92	ACU(T)	112	2.49
AUC(I)	17	0.08	ACC(T)	4	0.09
AUA(M)	249	1.88	ACA(T)	63	1.40
AUG(M)	16	0.12	ACG(T)	1	0.02
AAU(N)	217	1.92	AGU(S)	46	1.45
AAC(N)	9	0.08	AGC(S)	4	0.13
AAA(K)	82	1.86	AGA(S)	77	2.43
AAG(K)	6	0.14	AGG(S)	0	0.00
GUU(V)	66	1.76	GCU(A)	92	2.44
GUC(V)	0	0.00	GCC(A)	12	0.32
GUA(V)	82	2.19	GCA(A)	45	1.19
GUG(V)	2	0.05	GCG(A)	2	0.05
GAU(D)	64	1.88	GGU(G)	80	1.58
GAC(D)	4	0.12	GGC(G)	0	0.00
GAA(E)	72	1.95	GGA(G)	106	2.09
GAG(E)	2	0.05	GGG(G)	17	0.33

*: Stop codon

Table S11. The count of microsatellite-like sequence in the complete control regions of Chrysopidae.

microsatellite-like sequence	*Ankylopteryx gracilis*	*Apochrysa matsumurae*	*Chrysoperla nipponensis*	*Chrysopa pallens*	*Chrysoperla externa*	*Nothochrysa sinica*
$(TA)_2$	125	43	62	95	63	60
$(TA)_3$	54	11	19	34	14	25
$(TA)_4$	18	5	7	10	5	9
$(TA)_5$	10	4	4	4	5	5
$(TA)_6$	6	1	3	3	2	4
$(TA)_7$	4	1	2	2	0	2
$(TA)_8$	3	0	2	1	0	2
$(TA)_9$	3	0	0	1	0	1
$(AT)_2$	140	36	50	84	61	69
$(AT)_3$	45	7	17	24	8	21
$(AT)_4$	17	5	8	7	6	10
$(AT)_5$	9	3	4	3	5	4
$(AT)_6$	5	1	2	2	0	3
$(AT)_7$	5	1	2	2	0	2
$(AT)_8$	3	0	2	1	0	1
$(AT)_9$	3	0	0	1	0	0
$(TAA)_2$	43	10	13	12	11	8
$(TAA)_3$	8	2	2	1	0	1
$(ATT)_2$	10	5	6	7	10	4
$(TAT)_2$	9	7	12	15	14	9
$(TAT)_3$	2	1	2	4	5	1
$(ATA)_2$	31	11	13	16	19	8
$(ATA)_3$	10	2	0	1	1	1
$(TTAA)_2$	5	2	2	2	3	2
$(AATT)_2$	4	6	1	1	3	1
$(TTAAA)_2$	1	0	0	1	0	1
$(TTTAAA)_2$	2	0	0	0	1	0

Table S12. Nucleotide composition and skews in the complete control regions of Chrysopidae.

Species	T(U)	C	A	G	Total	A+T%	AT-Skew	GC-Skew
Ankylopteryx gracilis	43.93	8.15	45.45	2.46	3416.0	89.38	0.02	-0.54
Apochrysa matsumurae	45.14	10.43	41.38	3.05	1409.0	86.52	-0.04	-0.55
Chrysoperla nipponensis	41.08	8.76	45.66	4.50	1244.0	86.74	0.05	-0.32
Chrysopa pallens	40.47	8.66	45.56	5.30	1905.0	86.04	0.06	-0.24
Chrysoperla externa	42.80	7.97	45.02	4.21	1355.0	87.82	0.03	-0.31
Nothochrysa sinica	45.79	6.29	44.45	3.46	1271.0	90.24	-0.01	-0.29

Table S13. Nucleotide composition and skews in the rRNAs.

rRNA	Species	T(U)	C	A	G	Total	A+T%	AT-Skew	GC-Skew
rrnL	*Abachrysa eureka*	41.55	6.04	41.62	10.79	1307.0	83.17	0.00	0.28
	Ankylopteryx gracilis	42.23	5.99	41.62	10.16	1319.0	83.85	-0.01	0.26
	Apochrysa matsumurae	39.57	6.24	42.54	11.64	1314.0	82.12	0.04	0.30
	Chrysopa pallens	41.25	6.57	40.49	11.69	1309.0	81.74	-0.01	0.28
	Chrysoperla externa	40.00	6.41	41.83	11.76	1310.0	81.83	0.02	0.29
	Chrysoperla nipponensis	40.55	6.50	41.09	11.86	1307.0	81.64	0.01	0.29
	Italochrysa insignis	40.28	6.30	41.89	11.53	1301.0	82.17	0.02	0.29
	Leucochrysa pretiosa	41.37	6.06	41.37	11.20	1303.0	82.73	0.00	0.30
	Nothancyla verreauxi	41.60	6.41	40.53	11.45	1310.0	82.14	-0.01	0.28
	Nothochrysa sinica	41.09	5.99	41.93	10.99	1319.0	83.02	0.01	0.29
	Nothochrysa sp.	40.40	6.04	42.54	11.02	1307.0	82.94	0.03	0.29
	Parankylopteryx sp.	41.50	5.99	42.03	10.47	1318.0	83.54	0.01	0.27
rrnS	*Abachrysa eureka*	41.47	6.72	39.92	11.89	774.0	-0.02	18.60	0.28
	Ankylopteryx gracilis	40.77	6.32	41.55	11.35	775.0	0.01	17.68	0.28
	Apochrysa matsumurae	39.43	6.70	40.98	12.89	776.0	0.02	19.59	0.32
	Chrysopa pallens	41.21	6.93	40.31	11.55	779.0	-0.01	18.49	0.25
	Chrysoperla externa	40.41	7.08	40.67	11.84	777.0	0.00	18.92	0.25
	Chrysoperla nipponensis	39.69	7.22	40.46	12.63	776.0	0.01	19.85	0.27
	Italochrysa insignis	40.88	6.73	40.75	11.64	773.0	0.00	18.37	0.27
	Nothochrysa sinica	39.92	6.89	41.33	11.86	784.0	0.02	18.75	0.27
